# Urothelial Carcinoma Arising on a Functional Kidney Graft

**DOI:** 10.3390/biomedicines13051118

**Published:** 2025-05-06

**Authors:** Oana Moldoveanu, Cătălin Baston, Bogdan Sorohan, Lucas Discălicău, Cristian Mirvald, Oana Mădălina Baston, Ioanel Sinescu

**Affiliations:** 1 Department of Urology, Carol Davila University of Medicine and Pharmacy, 020021 Bucharest, Romania; catalin.baston@umfcd.ro (C.B.); lucas.discalicau@drd.umfcd.ro (L.D.); cristian.mirvald@umfcd.ro (C.M.); ioanel.sinescu@umfcd.ro (I.S.); 2 Center of Surgical Urology and Kidney Transplantation, Fundeni Clinical Institute, 022328 Bucharest, Romania; bogdan.sorohan@umfcd.ro; 3 Department of Nephrology, Carol Davila University of Medicine and Pharmacy, 020021 Bucharest, Romania; 4 Department of Radiology, Carol Davila University of Medicine and Pharmacy, Medical Imaging and Interventional Radiology, 020021 Bucharest, Romania; madalina.baston@umfcd.ro; 5 Department of Radiology, Medical Imaging and Interventional Radiology, Dr. Carol Davila Central Military Emergency University Hospital, 010242 Bucharest, Romania

**Keywords:** urothelial carcinoma, kidney transplant, allograft tumor, donor-derived malignancy, bladder cancer, BCG instillations

## Abstract

**Introduction**: Kidney transplant recipients present a higher risk of developing malignancies than the general population. Malignancies represent the third leading cause of death for kidney transplant recipients. There is an increased risk of developing urothelial carcinoma among kidney transplant recipients, but it is not as high as the risk of renal cell carcinoma, which is the most common urologic malignancy. Although the bladder is the most common location for urothelial carcinoma, urothelial carcinomas of the upper tracts of the native kidneys and the allograft are also reported. The estimated incidence of urothelial carcinomas arising on kidney grafts is 0.019%. **Case report:** We present a case of a kidney transplant recipient who developed non-muscle-invasive bladder cancer 10 years after the transplant. This was successfully treated with TURBT (transurethral resection of the bladder tumor) and BCG (bacillus Calmette–Guerin) instillations. Two years later, this patient developed metastatic urothelial carcinoma of the allograft. **Discussion:** Nephroureterectomy of the transplant with bladder preservation after BCG treatment, no systemic chemotherapy, and cessation of immunotherapy were the treatments of choice in this case. Local oncologic control and spontaneous complete regression of pulmonary metastasis were obtained at a 2-year follow-up. **Conclusions:** With this case, we emphasize the fact that managing urothelial carcinomas in kidney transplant recipients is a provocative challenge for surgeons, nephrologists, and oncologists, as there are no treatment guidelines or protocols.

## 1. Introduction

Urothelial carcinoma (UC) represents a significant clinical challenge, particularly among kidney transplant recipients, who face significant risk of developing higher-stage and higher-grade malignancies due to immunosuppressive therapies [1,2,3,4,5,6,7]. The incidence of urothelial carcinomas in this population is approximately three times higher than in the general populace. Therefore, careful surveillance and management strategies are needed [8]. While most post-transplant UCs are located in the bladder, upper tract urothelial carcinoma (UTUC) of the kidney graft is exceedingly rare. It is mostly presented in case reports and has an estimated incidence of just 0.019% in the kidney transplant population [1,3,4,8,9]. There are no specific guidelines for diagnosing and treating this type of malignancy in kidney transplant recipients [8,10,11,12]. We present a case of a male patient who received a kidney transplant from a deceased donor. He developed a urothelial carcinoma of the bladder 10 years after kidney transplantation, which was successfully treated with TURBT and BCG. Notably, 2 years after the initial diagnostic, he developed locally advanced graft upper tract urothelial carcinoma and pulmonary metastasis. To our knowledge, this is the first reported case of graft UTUC following BCG treatment for bladder UC in a transplant recipient. Through this case, we aim to highlight important insights into the clinical presentation, management complexities, and prognostic implications associated with urothelial carcinoma in kidney transplant recipients, underscoring the urgent need for personalized treatment protocols and robust follow-up strategies in this vulnerable patient cohort.

## 2. Case Presentation

**Figure 1:**
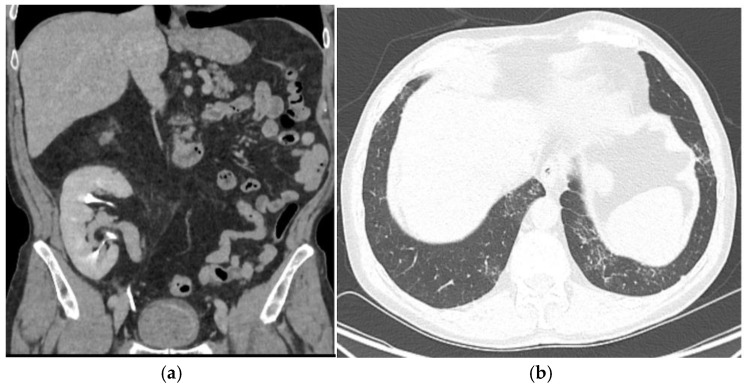
A 1-year follow-up CT scan showing no bladder recurrence, no graft lesion, and no pulmonary metastasis. (**a**): CT scan showing no bladder recurrence or graft lesion (axial view). (**b**): CT scan showing no pulmonary metastasis(axial view).

A 67-year-old male patient, a non-smoker who received a kidney transplant from a deceased donor, with no record of malignancy, in the right iliac fossa in 2010, presented in December 2020 due to recent gross hematuria. According to his medical history, the cause of his end-stage kidney disease was obstructive uropathy due to nephrolithiasis. Also, a left native nephrectomy was performed for pyonephrosis. The immunosuppression regimen following the transplant consisted of tacrolimus (targeted tacrolimus level: 5–7 ng/mL), mycophenolate mofetil, and prednisone. After transplantation, the baseline eGFR was 74 mL/min/1.73 m. He had no history of infection with the BK virus (BK polyomavirus). Laboratory studies showed a hemoglobin level of 14.0 g/dL, a white blood cell count of 8120/μL, and an eGFR (glomerular filtration rate) of 78 mL/min/1.73 m. The urine culture was negative. Urinary cytology was performed on all presentations in our hospital and returned negative for malignant cells in all instances. An ultrasound evaluation revealed the presence of three bladder tumors measuring 10–12 mm each, which were confirmed by cystoscopy on the left lateral vesical wall. TURBT was performed with no perioperative complications. A pathology exam revealed multiple pTaN0M0 low-grade urothelial carcinomas. BCG induction was initiated and maintained for 1 year with isoniazid protection treatments within 5 days of the procedures. No modifications of the immunosuppression regimen were made before or after TURBT. Also, the immunosuppression regimen was maintained during and after intravesical BCG therapy. A CT scan 1 year after TURBT showed no bladder recurrence, graft tumor, or metastasis (Figure 1).

**Figure 2:**
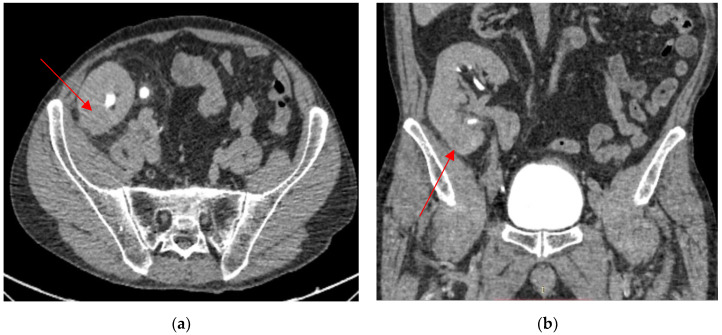
An inflammatory lesion localized in the lower pole of the kidney graft (lesion size: 29/26 mm). Axial view (**a**). Coronal view (**b**).

Six months after stopping BCG, the patient presented with fever, pain in the right fossa, and microscopic hematuria. Urinalysis revealed leucocituria and gross hematuria, but urine cultures were negative. The clinical suspicion of graft pyelonephritis was confirmed by a CT scan, which described a 29/26 mm inflammatory lesion. The decision to initiate antibiotic therapy was purely clinical, based on the symptoms, the urinalysis, and the appearance on the CT scan. He received 750 mg of Levofloxacin for 10 days, with complete remission of the symptoms. Cystoscopy was not performed during this visit and was scheduled after 3 months. It showed no recurrence. The inflammatory response under prednisone was normal, and a urine culture was negative. However, an ultrasound and a CT scan showed progression of the lower pole lesion of the allograft (Figure 2).

**Figure 3:**
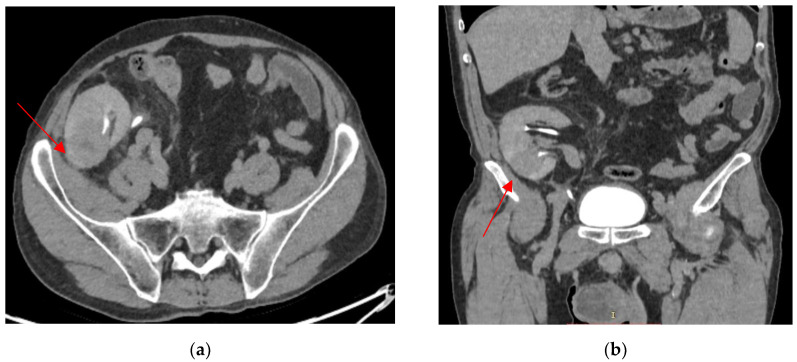
The progression of the lower pole kidney graft inflammatory lesion (lesion size: 41/38 mm). Axial view (**a**). Coronal view (**b**).

Twenty-four months after TURBT, he was admitted for gross hematuria. Another contrast-enhanced CT scan was performed, and it revealed progression of the lower pole graft lesion, with extension in the lower calyx and perinephric fat invasion. There was no bladder tumor recurrence (Figure 3), but there was a de novo solitary 20/19 mm lower left pulmonary lesion (Figure 3a). Cystoscopy was normal, and ureteroscopy could not be performed due to an inability to access the bladder via ureteric implantation. BCG pyelonephritis was suspected due to intravesical therapy exposure and was confirmed by urinary tests.

**Figure 4:**
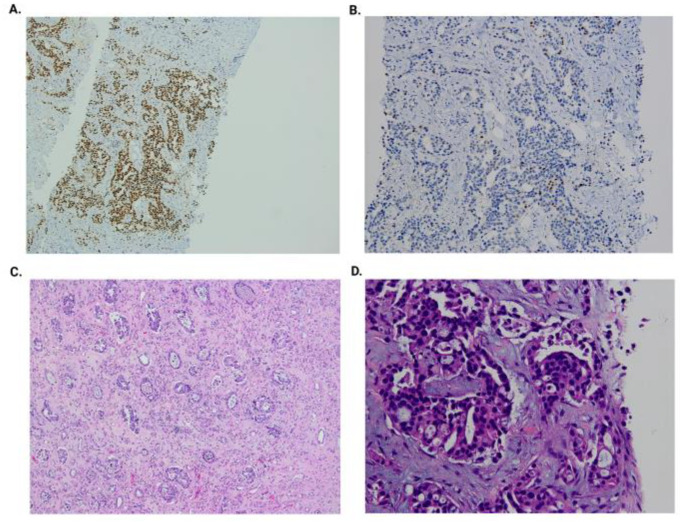
Kidney graft biopsy: GATA3-positive immunohistochemistry (**A**); p63-positive immunohistochemistry (**B**); graft nephroureterectomy (H&E at 200×) and high-grade urothelial carcinoma (LVI+, PNI+, and LI+) (**C**); and graft biopsy (H&E at 400×) with hyperchromatic and moderate pleomorphic nucleoli, irregular contours, and focal ischemic-type necrosis (**D**).

A kidney graft biopsy was performed, and the pathology exam confirmed the diagnosis of a urothelial malignant process (immunohistochemistry: CK7+, GATA3+, and p63+). Nephroureterectomy of the allograft and bladder cuff resection were performed. Hemodialysis was initiated, and immunosuppression was stopped. The pathology exam result was pT4N0M1 high-grade (LVI+, VI+, and PNI+) pelvicalyceal urothelial carcinoma with sinus and perinephric fat invasion and psoas muscle involvement (Figure 4).

**Figure 5:**
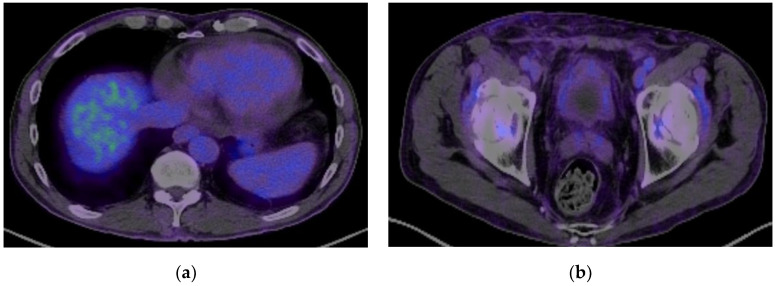
A PET-CT scan showing remission of the pulmonary metastasis (**a**) and no recurrence of the malignant process (**b**).

**Figure 6:**
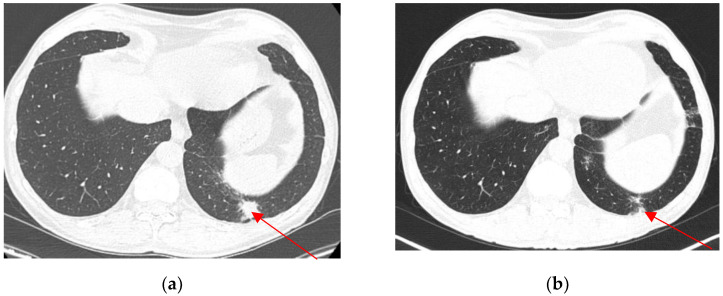
Pulmonary metastasis (20/19 mm) (axial view) before nephroureterectomy (**a**). Regression of lower pulmonary pole metastasis (axial view) 3 months after nephroureterectomy (**b**).

Three months after the operation, 18-FDG PET/CT showed no local recurrence, no lymph nodes, no metastasis, and regression of the pulmonary lesion (Figure 5 and Figure 6b). The oncology team decided not to treat with adjuvant chemotherapy and scheduled regular follow-ups with cystoscopy at 3, 6, and 12 months and a contrast-enhanced CT scan at 12 months. The tests were normal at the 1-year follow-up.

## 3. Discussion

As far as we know, this is the first case reported in the literature of a kidney transplant recipient who developed graft upper tract urothelial carcinoma detected after BCG treatment of urothelial carcinoma of the bladder.

Kidney transplant recipients have a higher risk of developing malignancies than the general population. The risk of developing urothelial carcinoma is 3-fold higher in this population but varies according to the geographic region. In East Asia, the risk of developing UC is 14 times higher, mostly affecting the upper tracts of the native kidneys. This is thought to be due to the consumption of Chinese herbs containing aristolochic acid [8]. Patients with end-stage renal disease who undergo kidney transplants are more likely to develop bladder cancer than non-kidney transplant patients with ESRD. Kidney transplant represents an independent risk factor for developing bladder cancer due to multivariate factors, including immunosuppression [1,3,4,9,13].

Upper tract urothelial carcinoma (UTUC) is a very rare type of cancer that affects transplanted kidneys. The first case of urothelial carcinoma in a renal graft was reported in 1993 by Lawrence [14], and most of the subsequent data came from case reports. A retrospective French national multicenter study, which collected data from 32 transplant centers, found 11 patients with graft UTUC out of 56,806 kidney transplant recipients, resulting in a very low incidence of 0.019% [8].

The most common risk factors for urothelial carcinoma are BKV and HPV infections, a history of smoking, aristolochic acid exposure, and analgesic abuse [10,11,12]. The duration of dialysis prior to a transplant has not been detected as a risk factor, though it is a risk factor for renal cell carcinoma in kidney transplant recipients [10]. In this case, no particular risk factor was identified and the donor-derived origin was not evaluated by genetic tests.

Compared to the general population, urothelial carcinoma in kidney transplant recipients has an inferior prognosis and lower survival rates. This might be due to the younger age at diagnosis and the advanced stage and higher grade at presentation [15].

The most common symptom at presentation for patients with urothelial carcinoma is hematuria. In kidney transplant patients, microscopic or macroscopic hematuria might indicate an underlying malignant cause and requires further investigations. Kim et al. found in a retrospective study that approximately 27% of kidney transplant recipients investigated for microscopic hematuria were diagnosed with a urologic malignancy [15,16,17].

For non-muscle-invasive bladder tumors, resection of the tumor, most often through TURBT, is the treatment of choice for bladder UC in kidney transplant recipients [18]. According to prognostic factor risk groups, intravesical BCG instillations are required to control local recurrence of bladder tumors in non-kidney transplant recipients [19]. Intravesical treatment with BCG in kidney transplant patients was controversial and considered inappropriate due to concerns related to safety and efficacy in an immunosuppressed population [20]. Small cohort studies have evaluated the efficacy and safety of intravesical BCG in kidney transplant patients. Similar oncological results were found for kidney transplant recipients and non-immunocompromised populations, contrary to the initial assumption that immunosuppression may reduce the effect of BCG [18,21,22]. In terms of safety, one case of mycobacterium infection leading to death [23] and one case of subacute interstitial pneumonitis [22] were reported after BCG instillations. In most cases, no severe reactions were reported, regardless of whether peri-instillation TB therapy with isoniazid, rifampicin, or ciprofloxacin was administered. Despite these findings, intravesical BCG is an uncommon treatment in kidney transplant recipients. In our case, 3 years after the initial resection, good oncologic control of the bladder was observed with no adverse reactions. An allograft biopsy excluded the initial suspicion of pyelonephritis caused by dissemination of mycobacterium and confirmed urothelial carcinoma of the kidney graft.

A large multicenter retrospective study was conducted to assess the predictors of the development of UTUC in non-kidney transplant patients with non-muscle-invasive bladder cancer treated with BCG therapy. The results showed that 6.1% of the patients treated with BCG developed an upper urinary tract tumor in the follow-up period. The risk factors for developing UTUC identified by the study were the BCG Connaught strain, multiple bladder tumors, and tumor recurrence [24,25]. In our case, taking into account the kidney transplant status of the patient, we can assume that multiple low-stage and low-grade bladder tumors represent a risk factor for developing UTUC in the kidney graft. Another presumption is that the urothelial carcinoma initially developed in the allograft, sustained by the higher-grade UTUC, and did not affect the native upper tract urothelium or the graft ureter. However, at the time of the bladder cancer diagnosis and at the CT scan performed during the 1-year follow-up there was no visual evidence of the graft UTUC. This might raise the question of whether in this case a late donor-derived malignancy can be incriminated, also taking into account the spontaneous remission of metastasis after nephroureterectomy and withdrawal of immunosuppression [8,12,26,27]. Controversial data from the literature indicate that donor-derived malignancies appear in the first 2 years after kidney transplants and are found in low stages with low grades [28], although there is one case report of donor-derived UC of the pelvis with a pT3N0M0 kidney graft and a pT1N0M0 native bladder 16 years post-kidney transplant (confirmed through dual-color fluorescent in situ hybridization (FISH)) [29].

There are no guidelines for the treatment of upper urinary tract urothelial carcinomas in kidney transplant recipients due to a lack of data that only come from case reports and small case series. Urothelial carcinoma of the allograft requires either resection and reconstruction of the urinary tract or nephroureterectomy and lymphadenectomy, as for the non-kidney transplant population [8].

For locally advanced or metastatic renal transplant urothelial carcinomas, graft nephrectomy and adjuvant chemotherapy with paclitaxel/cisplatin/gemcitabine demonstrated good local oncologic control and complete remission of pulmonary metastasis at 54 months of follow-up, despite tumor progression after two courses of neoadjuvant treatment with cisplatin and gemcitabine [30,31]. The decision to initiate adjuvant chemotherapy must take into account its high toxicity rates, and in the case of spontaneous regression of metastasis after kidney transplant nephrectomy, adjuvant chemotherapy can be stopped or delayed [32,33]. Complete responses were obtained after administration of immune checkpoint inhibitors, pembrolizumab, and atezolizumab, as a second-line therapy for metastatic urothelial carcinoma of the allograft, in two cases (reported at 21 months for pembrolizumab and 12 months for atezolizumab) [34,35].

In this case of a metastatic tumor of the allograft, no conservative treatment could have provided an oncologic benefit and nephroureterectomy was the treatment of choice. The rapid regression of the pulmonary metastasis led the oncology team to refrain from administering platinum-based chemotherapy and to intensify the follow-up program. The origin of the donor-derived malignancy is also an important issue to consider.

An important issue in this case is the feasibility and the appropriate timing of another transplant. According to the American Society of Transplantation (AST), no more than 6 months of waiting time is recommended for superficial bladder cancer, while a waiting period of at least 2 years is recommended between cancer remission and kidney transplantation for invasive bladder cancer after cystectomy [36]. There is no specific recommendation for patients with no local recurrence of bladder cancer, progression to locally advanced and metastatic urothelial carcinoma of the kidney graft nephroureterectomy, and remission of pulmonary metastases. Further reports in the literature and studies must evaluate whether re-transplantation is the optimal treatment for these patients to prevent long-term complications of dialysis.

## 4. Conclusions

This case of urothelial carcinoma management in a kidney transplant recipient provides significant insights into the elevated cancer risk in kidney transplant recipients. Effective surveillance and immediate intervention are crucial due to tumor complexities and treatment responses. In this case, graft nephroureterectomy in association with cessation of immunosuppression led to complete cancer remission. Although intravesical BCG therapy is controversial for kidney transplant recipients, for our patient, no bladder recurrence was identified, allowing preservation of the bladder. The tumorigenesis of the graft UTUC in this case is debatable, as it is not possible to exclude a donor-derived origin or the pre-existence of the UTUC at the time of the UC of the bladder diagnosis. Further studies should be conducted to elaborate diagnostic and therapeutic guidelines for kidney transplant patients diagnosed with UTUC of the kidney grafts, taking into consideration the balance between the oncological outcome and maintaining graft function, and to establish the optimal time for re-transplantation for patients after graft nephroureterectomy.

## Data Availability

The original contributions presented in this study are included in the article. Further inquiries can be directed to the corresponding author.
