# Correction: Moldoveanu et al. Urothelial Carcinoma Arising on a Functional Kidney Graft. *Biomedicines* 2025, *13*, 1118

**DOI:** 10.3390/biomedicines13102383

**Published:** 2025-09-28

**Authors:** Oana Moldoveanu, Cătălin Baston, Bogdan Sorohan, Lucas Discălicău, Cristian Mirvald, Oana Mădălina Baston, Ioanel Sinescu

**Affiliations:** 1Department of Urology, Carol Davila University of Medicine and Pharmacy, 020021 Bucharest, Romania; catalin.baston@umfcd.ro (C.B.); lucas.discalicau@drd.umfcd.ro (L.D.); cristian.mirvald@umfcd.ro (C.M.); ioanel.sinescu@umfcd.ro (I.S.); 2Center of Surgical Urology and Kidney Transplantation, Fundeni Clinical Institute, 022328 Bucharest, Romania; bogdan.sorohan@umfcd.ro; 3Department of Nephrology, Carol Davila University of Medicine and Pharmacy, 020021 Bucharest, Romania; 4Department of Radiology, Carol Davila University of Medicine and Pharmacy, Medical Imaging and Interventional Radiology, 020021 Bucharest, Romania; madalina.baston@umfcd.ro; 5Department of Radiology, Medical Imaging and Interventional Radiology, Dr. Carol Davila Central Military Emergency University Hospital, 010242 Bucharest, Romania

## References

The original publication [1] cited a retracted article: “2. Zhang, B.; Shen, C.; Han, W.K.; Yu, W. Comparison of Clinicopathologic Characteristics of Urothelial Carcinoma between Patients after Renal Transplantation and on Dialysis. *Transplantation* **2014**, *98*, 552–556.” This reference has now been deleted. With this correction, the order of some references has been adjusted accordingly. The authors state that the scientific conclusions are unaffected. This correction was approved by the Academic Editor. The original publication has also been updated.

## References

[1] Moldoveanu O., Baston C., Sorohan B., Discălicău L., Mirvald C., Baston O.M., Sinescu I. (2025). Urothelial Carcinoma Arising on a Functional Kidney Graft. Biomedicines.

